# Supplementary material for: Glucose-coated superparamagnetic iron oxide nanoparticles prepared by metal vapor synthesis can target GLUT1 overexpressing tumors: In vitro tests and in vivo preliminary assessment
Source: PLoS One. 2022 Jun 15;17(6):e0269603. doi: 10.1371/journal.pone.0269603 (PMC9200296; doi:10.1371/journal.pone.0269603)
Supplement: Attachment — Submitted filename: response to reviewers.docx [file pone.0269603.s001.docx]

General requests

In the Methods section we have provided additional information regarding the well being animals.

REVIEWER 1

1)We have re-edited manuscript by an experienced mother tongue ( “**E4ACEnglish for Academics**” sas di Adrian John Wallwork Via Carducci 9, 56127 Pisa Tel. 340 7888 304 (no fax) adrian.wallwork@gmail.com p.iva / c.f. 01923950503 REA PI – 166038)

2)We have re-edited the introduction: We think to realize that Reviewer asks for a starting with a more generical setting regarding the nano-therapy for cancer and for this reason we have modified the beginning of the introduction and quoted the paper on auto-assembled peptides as suggested. Moreover we have edited and streamlined the whole introduction deleting some parts.

3) We have added the references asked by the Reviewer and we have added some new references.

4) We have merged the figures as suggested

5) We have realized that the graphs hadn’t a not so good quality so we have completely re-done color graphs

6) We have corrected the text

7) We have added an illustration as requested

8)We have added a graphical abstract

9) We have added the reference and implemented the discussion following it

10) we have deleted old references as suggested

In yellow the new we parts and in reds the deleted parts throughout the “ not cleaned version “ of the manuscript

REVIEWER 2

According to the reviewer comment, the zeta potentials of the glucose coated superparamagnetic iron oxide nanoparticles at different pH values (7, 8.3, 3) have been measured. The results have been summarized in the table reported in Fig. 2 F and included into the manuscript according to that reported below. We have reported that the colloid were stable at room temperature.

In yellow the new we parts and in reds the deleted parts throughout the “ not cleaned version “ of the manuscript

PS we have inserted in the manuscript how we measured the volume of the tumor according to the last query
